# Plasma levels of microRNA are altered with the development of shock in human sepsis: an observational study

**DOI:** 10.1186/s13054-015-1162-8

**Published:** 2015-12-18

**Authors:** Andrew J. Goodwin, Changrun Guo, James A. Cook, Bethany Wolf, Perry V. Halushka, Hongkuan Fan

**Affiliations:** Division of Pulmonary, Critical Care, Allergy and Sleep Medicine, Medical University of South Carolina, 96 Jonathan Lucas Street, Charleston, SC 29425 USA; Department of Neurosciences, Medical University of South Carolina, 171 Ashley Avenue, Charleston, SC 29425 USA; Department of Public Health Sciences, Medical University of South Carolina, 135 Cannon Street, Charleston, SC 29425 USA; Departments of Pharmacology and Medicine, Medical University of South Carolina, 68 President Street, Charleston, SC 29425 USA

**Keywords:** Sepsis, Shock, MicroRNA, Endothelial cell

## Abstract

**Background:**

Endothelial dysfunction plays a critical role in the development of sepsis-related organ failure; however, the mechanisms that govern its development are not fully understood. Endothelial progenitor cells (EPCs) reduce vascular leak and organ failure in experimental sepsis while modulating plasma expression of microRNA (miRNA). MicroRNAs are small, noncoding segments of RNA that regulate gene expression and are known to modulate endothelial cell function and inflammatory signaling pathways. We hypothesized that miRNA may play an etiologic role in the endothelial dysfunction of sepsis and that their extracellular expression levels would be altered in those with shock.

**Methods:**

Thirteen miRNAs were identified by literature search and analysis of the contents of human EPC-derived exosomes using real-time PCR. Plasma samples were obtained from patients within 24 hours of their admission to ICUs with severe sepsis (n = 62) and from healthy controls (n = 32) and real-time PCR was used to measure the expression of the candidate miRNAs. The Wilcoxon rank sum test was used to compare expression levels of the 13 candidate miRNAs in septic patients with (n = 29) and without (n = 33) shock while logistic regression was used to determine the area under the curve for associations between miRNA expression and shock. Bioinformatic analyses using miRNA databases were performed to identify pathways and gene targets of differentially expressed miRNA with potential relevance to sepsis-related shock.

**Results:**

MiRNA-34a expression was significantly increased in the group who developed shock (*p* = 0.03) while miR-15a and miR-27a expressions were significantly decreased in this group (*p* = 0.006 and 0.03, respectively). The combined expression of these three miRNAs predicted shock with an area under the curve of 0.78 (95 % CI 0.66–0.90). In silico analyses predict that these three miRNAs regulate genes involved in endothelial cell cycle, apoptosis, VEGF signaling, LPS-stimulated MAPK signaling, and nuclear factor kappa B signaling.

**Conclusions:**

The plasma levels of miRNA are altered in patients with severe sepsis complicated by shock and may offer prognostic value as well as insights into the mechanisms of endothelial dysfunction in sepsis.

## Background

Sepsis is a heterogeneous syndrome characterizing the body’s response to serious infection. Although a standard clinical definition has been proposed [1], its clinical course is highly variable with some patients experiencing a relatively benign illness and others progressing to shock and multi-organ failure [2]. Endothelial dysfunction is a key hallmark of this progression as its resultant vascular permeability leads to cardiovascular compromise as well as organ edema and failure. While several critical functions of the endothelium have been identified in sepsis [3–6], the biological underpinnings governing the development of endothelial dysfunction in this syndrome are incompletely understood and represent an important area of study in order to characterize the pathogenesis of septic shock.

MicroRNAs (miRNAs) are small (19–25 nucleotides) noncoding segments of RNA that regulate gene expression by binding to target mRNA and inhibiting their translation [7–9]. Cell-to-cell delivery of miRNAs through vesicular structures such as exosomes has been described in a variety of cell types including endothelial cells and has been shown to impact cellular function [10–13]. Our previous work demonstrated that treatment of murine sepsis with human endothelial progenitor cells (EPCs) improves survival while attenuating vascular leak and its resultant organ failure [14]. Endothelial progenitor cells release exosomes that contain miRNAs known to promote the homeostasis and barrier integrity of endothelial cells [14–17] and may be a mechanism by which EPCs modulate sepsis-induced vascular leak. Thus, a deeper understanding of the role that endothelial-relevant miRNAs may play in the pathogenesis of endothelial dysfunction and shock is warranted.

In the current study, we have demonstrated that 13 miRNAs that have known associations with sepsis [14, 18–31] are found inside EPC-derived exosomes. Further, we have examined the expression levels of these miRNAs in plasma collected from patients experiencing severe sepsis and assessed for associations with the development of shock and organ failure. We hypothesized that patients who experience shock and organ failure will express higher levels of miRNAs that could reduce endothelial barrier integrity through gene inhibition and, conversely, express reduced levels of miRNAs that could enhance endothelial barrier integrity.

## Methods

### Subject recruitment and sample acquisition

We screened all new intensive care unit (ICU) admissions at a single tertiary-care academic hospital from July 2013 to February 2015 for the presence of severe sepsis based on the American College of Chest Physicians/Society of Critical Care Medicine consensus definition [1]. Additional inclusion criteria included age ≥ 18 years and admission into the ICU within the previous 24 hours. We excluded immunocompromised patients as defined by: immunosuppressive medication use, leukopenia, current hematologic malignancy, and history of stem cell transplant, and excluded patients transferred in from other hospitals if they had spent > 24 hours in an ICU at the time of screening. Patients who had transitioned to comfort measures only at the time of screening were further excluded. Healthy control subjects were recruited through local advertising. Informed consent for study participation and publication of results was obtained from all research subjects or their legally authorized representatives.

Consenting subjects had blood drawn via venipuncture or from pre-existing intravascular catheters. Blood samples from septic patients were collected within 24 hours of admission to the ICU. Samples were centrifuged at 3400 × g for 10 minutes and the plasma supernatant was collected and stored in aliquots at -80 degrees Celsius. Demographic and clinical information from septic patients was abstracted from the electronic medical record including the source and type of infection as well as variables required to calculate acute physiology and chronic health evaluation (APACHE) II scores. Clinical outcomes including occurrence of shock (defined by vasopressor use), occurrence of acute kidney injury [Acute Kidney Injury Network (AKIN) criteria], occurrence of acute respiratory distress syndrome (ARDS) by the Berlin definition [32], ICU and hospital length of stay, discharge destination and vital status were similarly captured. Basic demographics were recorded from healthy controls.

### Human EPC isolation, culture and exosome analysis

Human EPCs were isolated from umbilical cord blood as previously described (Medina). Cells were cultured in EGM-2 medium (Lonza, Walkersville, MD, USA) containing 10 % of exosome-depleted fetal bovine serum (System Biosciences, Mountain View, CA, USA). Exosomes were isolated from EPC-conditioned medium using ExoQuick Exosome Precipitation Solution (System Biosciences) following the manufacturer’s instructions. MiRNAs were isolated by miRNeasy kits (Qiagen, Valencia, CA, USA) and analyzed by real-time polymerase chain reaction (RT-PCR).

### Candidate miRNA selection

Using the combination of MeSH terms for “sepsis” and “microRNA”, we searched MEDLINE for articles that describe associations between the miRNA expression and sepsis. Each article was reviewed and associated miRNA were recorded and then searched individually in conjunction with sepsis (e.g., “miR-146a” and “sepsis”) in order to identify any additional references that associate miRNA expression and sepsis. In order to focus the investigation on miRNA with a high likelihood of relevance, we considered only miRNAs with at least two published references associated with sepsis to be potential candidates for investigation. These candidates were then cross-referenced with miRNA expression data from an array performed on human EPC-derived exosomes. MiRNAs that were not measured or in very low abundance in these exosomes were excluded, while the remaining miRNAs comprised the list of candidate miRNAs included in the analysis.

### Real-time PCR

Plasma samples from septic and healthy control subjects and EPC-derived exosome contents each underwent real-time PCR analysis. Candidate miRNAs were isolated from plasma and exosome samples using miRNeasy serum/plasma kits and then amplified with miScript SYBR Green PCR kits and primers specific to each miRNA (Qiagen). The mean Cq of the healthy control subjects was used to normalize the Cqs for each miRNA. Delta Cqs were then converted to and presented as fold change compared to the healthy control mean expression values.

### Bioinformatic analysis

Ingenuity Pathway Analysis (Qiagen) was used to examine the canonical pathways in which differentially expressed miRNAs in shock were involved. The software uses Fisher’s exact test to determine the statistical probability that a given canonical pathway is modulated by the inputted miRNA. Significant pathways are represented by the negative log of the *p* value; we a priori chose to only present pathways with a *p* value ≤ 0.001 corresponding to a negative log value of four. Subsequently, the miRNA target databases TargetScan [34] and MiRanda [35] were analyzed and both experimentally validated and predicted gene targets involved in canonical pathways of relevance were identified and used to manually construct a gene network for the differentially expressed miRNA.

### Statistical analysis

Baseline characteristics of human subjects were compared between the healthy control, septic patients without shock, and septic patients with shock groups using analysis of variance (ANOVA) for continuous variables and the chi-squared test for categorical variables. Continuous clinical characteristics were compared between septic patients who experienced shock and those who did not using the Student’s *t* test or Wilcoxon rank sum test as appropriate while categorical variables were compared using the chi-squared or Fisher exact test. MiRNA expression is presented as a continuous fold change variable and compared between the two sepsis groups using the Wilcoxon rank sum test. Additionally, miRNA expression was compared between groups defined by the presence or absence of acute kidney injury and ARDS as well as between groups defined by an APACHE II score of less than versus greater than or equal to 25. Correlation between miRNA levels and APACHE II scores was further assessed for using Spearman’s correlation. Area under the curve (AUC) was estimated for each miRNA based on univariate logistic regression models comparing the subjects who experienced shock and those who did not. A multivariable logistic regression was also developed to determine the predictive performance of a combination of the candidate miRNAs. The AUC for the most predictive single miRNA was compared to the AUC from the multiple logistic regression model using Delong’s test for comparing nested AUCs. Bootstrap confidence intervals based on 1000 bootstrap samples were also estimated for all AUCs. All analyses were conducted in SAS v. 9.3 (SAS Institute, Cary, NC, USA). All aspects of this study were approved by the Institutional Review Board at the Medical University of South Carolina.

## Results

A total of 228 patients with a clinical picture suggestive of severe sepsis were admitted to the medical or surgical ICUs during the study enrollment period. Of these, 12 (5 %) were deemed by the study team and the clinical team to not have sepsis after detailed review of the clinical history. Of the remaining 216 patients, 121 (56 %) were excluded because consent was unobtainable within the first 24 hours of presentation to an ICU. Further, 27 (13 %) patients were excluded for having an immunocompromised condition, and six (3 %) were excluded because they were receiving comfort measures only at the time of screening. The remaining 62 (29 %) were enrolled and included in the analysis in addition to 32 healthy control volunteers. Healthy control subjects were on average younger and more likely to be of white race (Table 1). Among septic subjects, those who developed shock were similar in age and race to those who did not but were more likely to be of male gender. The source of infection was similar between those who experienced shock and those who did not, as was the type of organism responsible for the sepsis. Subjects who experienced shock had no significant differences in their rates of mechanical ventilation, ARDS, or acute kidney injury as well as ICU or hospital lengths of stay; however, they did have a nonsignificant trend toward higher APACHE II scores at enrollment and a significantly higher mortality rate.

**Table 1:** Characteristics of study subjects

Variable	Healthy controls	No shock	Shock	*p* value
	n = 32	n = 33	n = 29	
Mean age (years) ± SD	40 ± 16	56 ± 18	58 ± 21	0.0002
Male gender (%)	15 (44)	15 (45)	22 (76)	0.03
White race (%)	28 (82)	19 (58)	16 (55)	0.01
Source of infection (%)	N/A			0.6
Urinary tract		11 (33)	9 (31)	
Pneumonia		9 (27)	12 (41)	
Intravascular device		5 (15)	2 (7)	
Other		7 (21)	6 (21)	
Organism (%)	N/A			0.28
Gram-negative bacteria		12 (36)	11 (38)	
Gram-positive bacteria		5 (15)	3 (10)	
Unknown		11 (33)	6 (21)	
Other		4 (12)	9 (31)	
Mechanical ventilation (%)	N/A	12 (36)	14 (48)	0.28
ARDS (%)	N/A	5 (15 %)	9 (7 %)	0.22
Acute kidney injury (%)	N/A	20 (61 %)	18 (62 %)	0.79
Mean APACHE II score ± SD	N/A	20.5 ± 7.4	23.5 ± 8.3	0.09
Median ICU LOS in days (IQR)	N/A	2 (7)	4 (4)	0.16
Median hospital LOS in days (IQR)	N/A	7 (11)	7 (12)	0.64
Death (%)	N/A	3 (9)	9 (31)	0.03

*SD* standard deviation, *N/A* not applicable, *ARDS* acute respiratory distress syndrome, *APACHE* acute physiology and chronic health evaluation, *ICU* intensive care unit, *LOS* length of stay, *IQR* interquartile range

### Expression of candidate miRNAs in human EPC exosomes

**Fig. 1:**
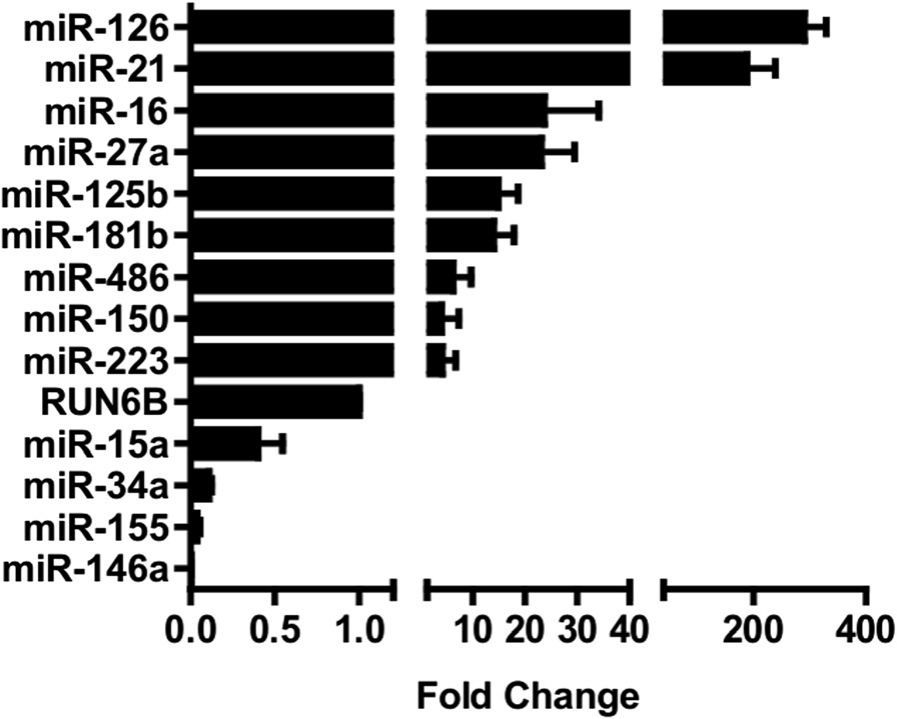
Expression of candidate miRNAs in human EPC-derived exosomes. Data normalized to the expression of RUN6B. *EPC* endothelial progenitor cells, *miRNA* microRNA

We identified 17 miRNAs with more than one published reference associating them with sepsis. Of these, four miRNAs were either not measured or expressed in very low levels in an array of human EPC-derived exosomes (data not shown) and were excluded from analysis while the remaining 13 miRNA were analyzed (Table 2). The expression levels of the candidate miRNA in human EPC-derived exosomes was determined by RT-PCR and displayed in Fig. 1. Expression levels are presented as fold changes from RNU6B expression as previously described [36, 37].

**Table 2:** Summary of candidate miRNA associated with sepsis

miRNA	Findings
miR-15a	Differentially expressed in adult and neonatal sepsis [18, 19]. Inhibits angiogenesis through direct targeting of VEGF and FGF [51]
miR-16	Differentially expressed in adult and neonatal sepsis [18, 19]. Regulates cell cycle entry, differentiation, and cytokine production in EPCs [52]
miR-34a	Plasma expression altered in murine sepsis [14]. Promotes endothelial senescence through targeting of SIRT1 [53]
miR-126	Plasma expression altered in murine sepsis [14]. Regulates the response of endothelial cells to VEGF through targeting of SPRED1 [15]
miR-27a	Upregulated in the lungs of septic mice [20, 21]. Knockdown reduced levels of TNF-α and IL-6 [21]
miR-150	Elevated in septic patients compared to patients with nonseptic SIRS [23]
	Lower levels of miR-150 associated with sepsis mortality [22]
miR-223	Elevated in septic patients compared to controls
	Expression level directly related to illness severity [25]
miR-181b	Inhibits NF-κB-mediated expression of VCAM1 in endothelial cells and reduces leukocyte influx into vascular endothelium [26]
miR-155	Upregulated in mice in response to systemic lipopolysaccharide. Targets several proteins in LPS signaling pathway [27]
miR-125b	Downregulated in mice in response to systemic lipopolysaccharide. Targets TNF-α [27]
miR-146a	Regulates IL-1β, IL-6, and TNF-α expression through targeting of IRAK1 in the NF-κB signaling pathway [28]
miR-486	Targets and inhibits NF-κB repressors resulting in its sustained signaling [29]
miR-21	Upregulated in mice in response to cecal ligation and puncture. Shown to facilitate the generation of myeloid-derived suppressor cells in late sepsis [30] Null miR-21 mice with higher mortality in LPS-peritonitis model [31]

*miRNA* microRNA, *VEGF* vascular endothelial growth factor, *FGF* fibroblast growth factor, *EPC* endothelial progenitor cells, *TNF-α* tumor necrosis factor alpha, *IL-6* interleukin-6, *SIRS* systemic inflammatory response syndrome, *NF-κB* nuclear factor kappa B, *LPS* lipopolysaccharide, *IL-1β* interleukin-1 beta

### Differential expression of miRNAs in shock

**Fig. 2:**
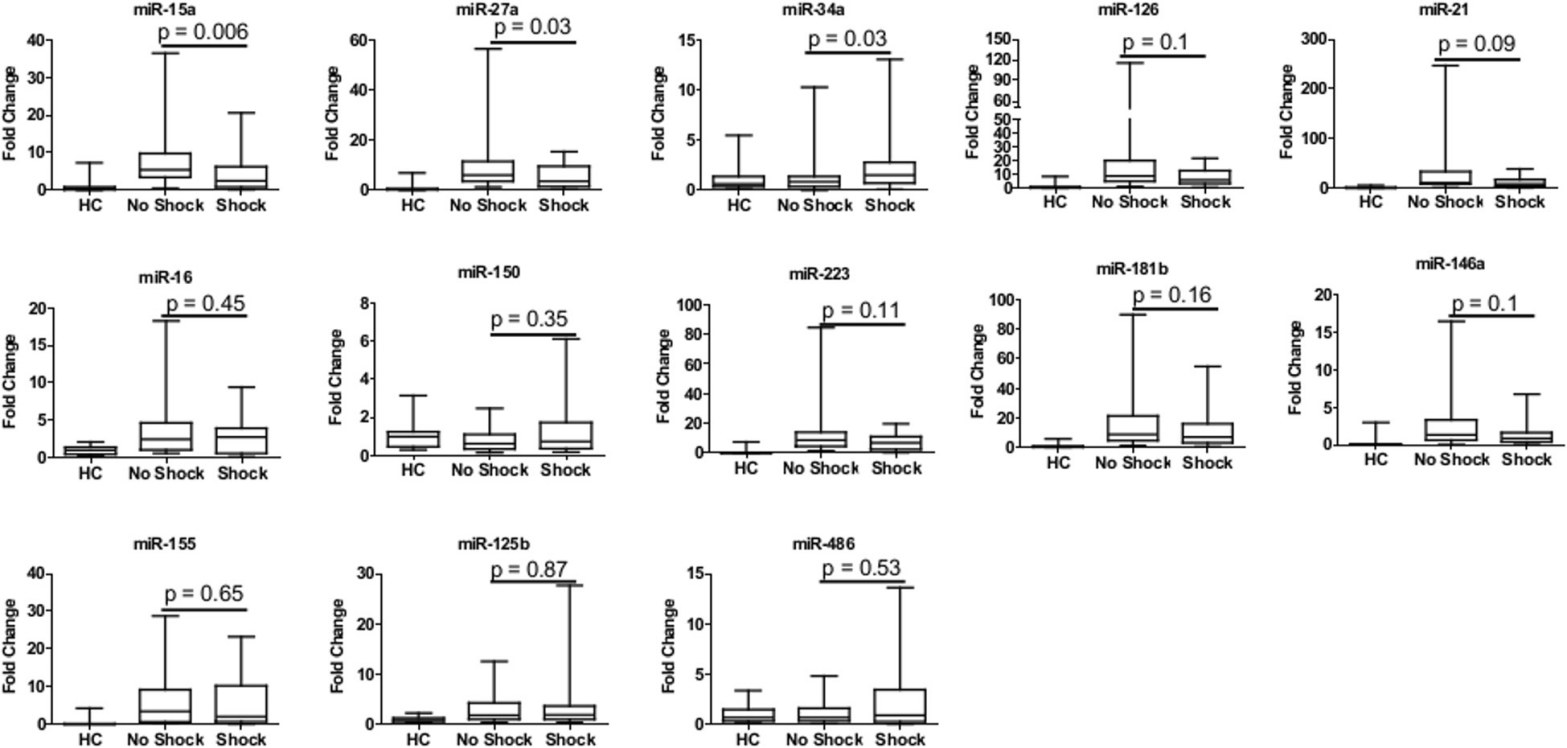
Plasma expression levels of miRNAs associated with sepsis in healthy controls (n = 32) and septic patients with (n = 29) and without shock (n = 33). Data presented as a fold change compared to the median value of the healthy control population for each miRNA. Box plots are displayed where the *horizontal bar* represents the median, the *box* represents the IQR and the *whiskers* represent the maximum and minimum values. Comparisons made by Wilcoxon rank sum test. *HC* healthy control, *miRNA* microRNA, *IQR* interquartile range

The expression levels of the candidate miRNAs were measured in the plasma from healthy controls as well as from septic patients with and without shock using RT-PCR. The mean expression level was calculated from the healthy control group for each miRNA and used to normalize the expression of that miRNA for each septic subject such that data is presented as a fold change in expression compared to the mean healthy control values. Expression levels were compared between healthy controls (n = 32) and the entire sepsis population (n = 62) as well as between septic subjects with (n = 29) and without shock (n =33). Sepsis patients demonstrated significantly higher plasma expression of each miRNA (all *p* < 0.05, data not shown) except miR-150 (*p* = 0.14) and miR-486 (*p* = 0.58) when compared to healthy controls. MiR-15a and -27a were significantly underexpressed in septic subjects who experienced shock compared to those who did not (*p* = 0.0062 and *p* = 0.03, respectively) while miR-34a was overexpressed (*p* = 0.03) in the shock group (Fig. 2). Patients in shock also exhibited nonsignificant trends toward reduced expressions of miR-21 and miR-126 compared to the subjects who did not experience shock (*p* = 0.9 and *p* = 0.1, respectively). There were no significant differences in the expression of any other miRNAs between the two sepsis groups.

In a separate analysis, miRNA levels were compared between septic subjects whose clinical course was complicated by acute kidney injury or ARDS and between subjects with high (≥25) or low (< 25) APACHE II scores. In total, 38 of 62 (61 %) subjects experienced acute kidney injury (defined as AKIN stage I or higher). Of these 38, only 18 (47 %) subjects had experienced vasopressor-dependent shock, demonstrating only modest overlap between the groups defined by the presence of shock and the presence of acute kidney injury. However, similar to the subjects who experienced shock, those who experienced acute kidney injury exhibited significantly lower levels of plasma miR-15a compared to those who experienced no acute kidney injury (*p* < 0.0001, data not shown). There were no significant differences in the expression levels of other miRNAs based on the presence or absence of acute kidney injury. Additionally, there were no associations or correlations between miRNA levels and presence of ARDS or APACHE II scores.

### The combination of differentially expressed miRNA predicts the development of shock

**Fig. 3:**
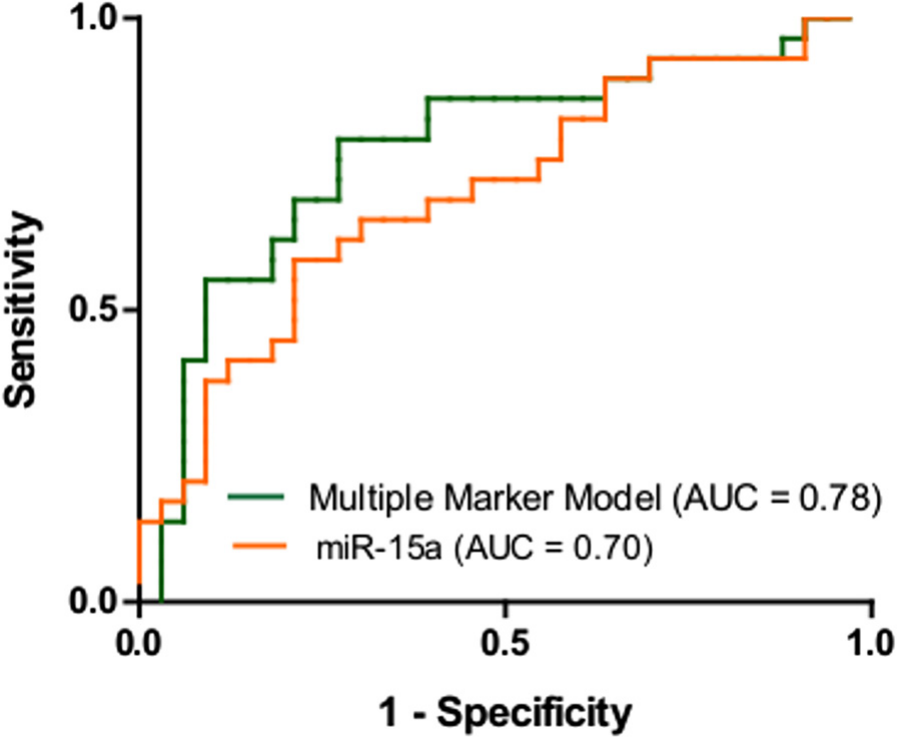
Receiver operating characteristics curve for miR-15a (*orange*) and for the multivariable logistic regression model including miR-15a, miR-27a, and miR-34a (*green*)

Logistic regression was used to determine associations between miRNA expression and shock. The area under the curve (AUC) values for individual miRNAs ranged between 0.66 and 0.70 (Table 3). MiR-15a was the most predictive single miRNA for distinguishing between sepsis patients with and without shock with an AUC of 0.70 [95 % confidence interval (CI) 0.57–0.84]. The final multiple marker model included miR-15a, -27a, and -34a. The receiver operating characteristics curves for miR-15a and for the multiple marker model are shown in Fig. 3. Although the AUC for the multiple marker model was larger than any individual marker [AUC = 0.78 (95 % CI 0.66–0.90)], there was not a significant difference in the AUC between it and miR15a (*p* = 0.090).

**Table 3:** Area under the curve (AUC) (95 % confidence interval) for individual miRNAs and for a multivariable logistic regression model

Marker	AUC
miR-27a	0.66 (0.52, 0.80)
miR-34a	0.67 (0.53, 0.80)
miR-15a	0.70 (0.57, 0.84)
miR-15a + miR-27a + miR-34a	0.78 (0.66, 0.90)

*miRNA* microRNA

### In silico analyses of the differentially expressed miRNA

**Fig. 4:**
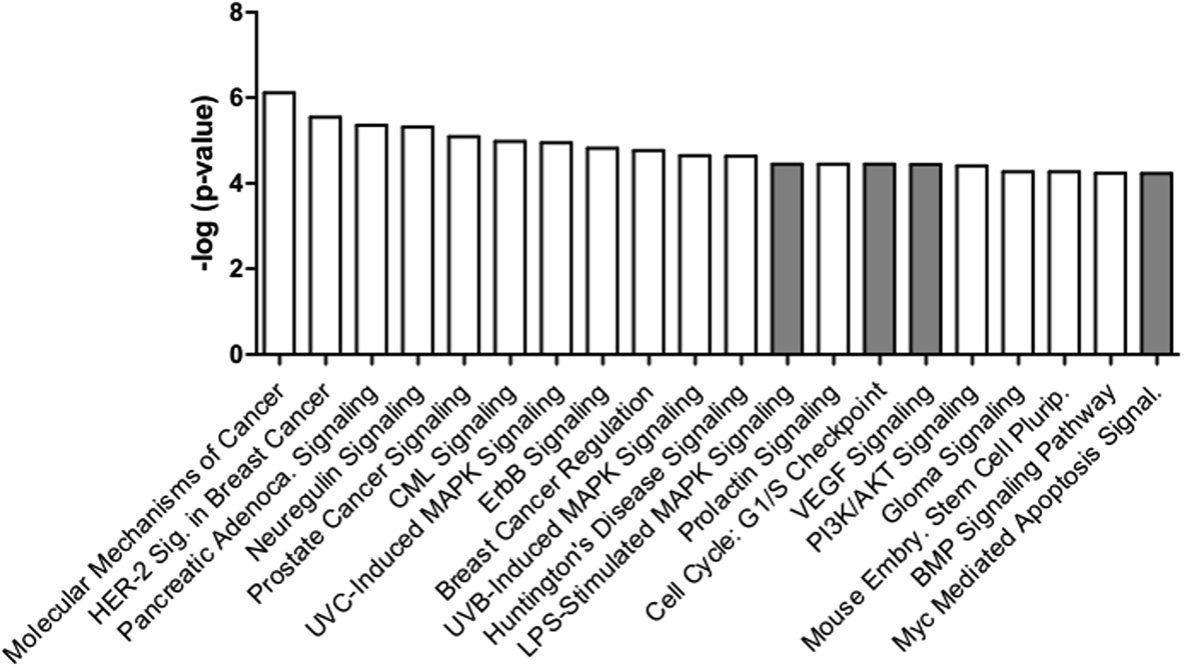
Canonical pathways predicted by Ingenuity Pathway Analysis to be modulated by miR-15a, -27a, and -34a. Data presented as the negative log of the *p* value of the Fisher’s exact test. *Shaded bars* represent pathways of potential relevance to endothelial dysfunction in sepsis

MiRNA-15a, -27a, and -34a were input into Ingenuity Pathway Analysis in order to predict the canonical pathways that may be differentially regulated in septic subjects who experienced shock. Twenty different pathways met the predetermined significance level of a negative log of the *p* value ≥ 4 (Fig. 4). Of these 20, four pathways appeared to be particularly relevant to the endothelial response to sepsis including LPS-stimulated MAP kinase signaling, cell cycle: G1/S checkpoint, vascular endothelial growth factor (VEGF) signaling, and myc-mediated apoptosis signaling.

**Fig. 5:**
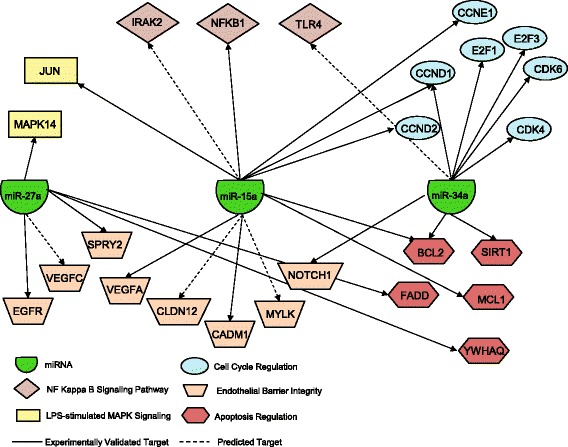
Gene target network of miR-15a, -27a, and -34a in pathways of relevance to endothelial dysfunction in sepsis. *Arrows* represent validated (*solid*) or predicted (*dashed*) targets of a miRNA. *miRNA* microRNA

Review of the TargetScan and miRanda databases identified 20 total genes that have been experimentally validated (*solid lines*) to be targets of miR-15a, -27a, or -34a and are also associated with pathways of potential relevance in sepsis (Fig. 5). These pathways include those identified in the canonical pathway analysis (cell cycle regulation, apoptosis, and LPS-stimulated MAP kinase signaling) as well as the nuclear factor kappa B (NF-κB) signaling pathway and a broader pathway of endothelial barrier integrity that includes VEGF signaling. Additional genes that are predicted targets of these miRNA and are included in these pathways are also displayed (*dashed lines*).

## Discussion

The results of this study demonstrate that the expression of candidate miRNAs with known associations with sepsis is altered in patients with septic shock. These data demonstrate that miR-15a, -34a, and -27a were all differentially expressed in patients whose course was complicated by shock and their combination was able to discriminate which patients had or developed shock with reasonable accuracy. In silico analyses predict that these three miRNAs modulate several pathways important to endothelial cell function including: cell cycle regulation, apoptosis, barrier integrity, lipopolysaccharide (LPS)-mediated signaling, and NF-κB signaling. Inside of these pathways, several gene targets have been identified that may represent key mediators in the development of endothelial dysfunction and progression to shock.

Since the initial discovery of extracellular miRNA, investigators have postulated that they provide a mechanism for cell-to-cell communication through manipulation of target cell gene expression [10]. MiRNAs are known to circulate in the plasma either enveloped in a vesicular structure such as an exosome, microvesicle or apoptotic body [10, 11, 38] or bound to protein such as argonaute 2 or high-density lipoprotein (HDL) [39, 40]. Endothelial cells can communicate with each other or with other cell types through the release of membrane-bound miRNAs, which are internalized by recipient cells leading to an alteration of gene expression [11, 12]. Thus, the expression patterns of extracellular miRNAs that modulate endothelial function and inflammatory signaling may provide key insights into mechanisms that contribute to endothelial activation and injury in the setting of severe sepsis. Through the identification of differentially expressed miRNAs in patients who developed shock, these data provide new directions of exploration into both mechanistic discovery and therapeutic intervention.

Although the data presented here represent only associations, there are several potential mechanisms by which the differential expression or miR-15a, -27a, and -34a could impact the development of endothelial dysfunction and shock. First, both miR-15a and miR-27a are known to or are predicted to target and inhibit genes that increase vascular permeability in the setting of sepsis including VEGFA, VEGFC and MYLK [41–44]. Therefore, reduced levels of circulating miR-15a and miR-27a could disinhibit these genes and contribute to the development of shock. Alternatively, miR-34a targets and inhibits BCL2 and SIRT1, which have both been identified as important negative regulators of apoptosis and cellular senescence in the endothelium [45, 46]. Thus, increased levels of circulating miR-34a could be contributing to the endothelial dysfunction in shock by augmenting apoptosis and senescence in response to cytokine stimuli. Finally, miR-15a may target several members of the NF-κB pathway and reduced circulating miR-15a could functionally augment NF-κB signaling leading to increased inflammatory cytokine production and increased endothelial injury.

In addition to providing mechanistic insight into the endothelial dysfunction of septic shock, the differential expression of miR-15a, -27a, and -34a may also offer prognostic biomarker capability. Unlike genomic DNA, which is static, RNA expression can dynamically change over healthy and diseased states and, thus, can provide real-time information regarding cellular function. And, as protein repression is a downstream consequence of miRNA function, changes in miRNA expression may precede changes in protein expression. Accordingly, miRNAs that are associated with the development of shock in patients with sepsis may provide an early glimpse into an individual’s risk at the time of initial presentation. Thus, rapid, automated analysis of plasma miRNAs could represent a useful prognostic biomarker with which to risk stratify patients with severe sepsis. Such a tool could be used to identify those at greatest risk for impending hemodynamic compromise potentially leading to more aggressive early intervention or changes in triage practices.

This study has limitations. As stated above, our data provide only associations between plasma miRNA levels and shock; therefore, we cannot determine if the differential expression of these miRNAs contributes to the development of shock or is a response to shock. In addition, our decision to examine a larger array of candidate miRNA limited our ability to confirm statistical significance with multiple comparisons testing. Future work will focus on validating the differential expression of miR-15a, miR-27a, and miR-34a in independent cohorts. Although all of the plasma samples from sepsis patients were collected within the first 24 hours of admission, these data are unable to determine the prognostic capabilities of these miRNA if measured immediately at the time of initial presentation. Future studies that examine miRNA expression at that time point will help to clarify their prognostic utility. Our data are further limited by our inability to identify the cellular source of the analyzed miRNA. A variety of cells exposed to plasma are known to release membrane-bound miRNA including lymphocytes, neutrophils, platelets, endothelial cells, and endothelial progenitor cells [11, 13, 14, 47, 48] and individual miRNAs are known to be released by numerous different cell types [49]. Further, comparisons between the miRNA expression in cells and their daughter exosomes has revealed markedly different miRNA expression patterns between the two compartments suggesting a selective export mechanism [10, 13, 50]. In combination, these characteristics create a significant challenge to determining the exact cellular source of circulating miRNA. Finally, this investigation is limited by the inability to study the downstream targets of the differentially expressed miRNAs in vivo. The vast majority of the known or predicted targets code for intracellular proteins, which would necessitate biopsy of tissue in order to analyze their expression in the endothelium in vivo. As patients admitted with severe sepsis uncommonly undergo tissue biopsy, this represents a limitation to our analysis. A notable exception is VEGFA, which is targeted by miR-15a and is secreted extracellularly. When analyzed, no association could be identified between VEGFA and miR-15a levels in subjects’ serum (data not shown). It is unclear from the current sample size if this lack of association is accurate or representative of type II error in setting of the statistical noise inherent to in vivo analysis of the critically ill. Since this also represents a single point in time in a disease with a protracted course, a time course study of serum miR-15a and VEGFA levels is warranted to definitively establish whether an association between the two exists.

## Conclusions

Altered plasma expression levels of miR-15a, -27a, and -34a are associated with the development of shock in patients with severe sepsis. These miRNA target cellular pathways that are critical to endothelial homeostasis including cell cycle regulation, apoptosis, cell layer permeability, and inflammatory response signaling. Future work will include earlier measurement of miRNA at the time of presentation to determine their prognostic capabilities as well as using in vitro and animal models of sepsis to clarify the potential roles that these miRNA and their targets play in the development of endothelial dysfunction and shock.

## Key messages

- Endothelial dysfunction is an important hallmark of the development of shock
- MicroRNA-15a, -27a, and -34a are differentially expressed in the plasma of septic patients who develop shock
- The expression of these microRNAs predict the presence of shock with very good accuracy
- In silico analyses predict that these microRNAs target and inhibit a number of genes that regulate the cell cycle, apoptosis, NF-κB signaling, LPS-stimulated MAP kinase signaling, and intercell permeability of endothelial cells.

## Abbreviations

ANOVA: Analysis of variance
APACHE: Acute physiology and chronic health evaluation
ARDS: Acute respiratory distress syndrome
AUC: Area under the curve
CI: Confidence interval
EPCs: Endothelial progenitor cells
HDL: High-density lipoprotein
ICU: Intensive care unit
LPS: Lipopolysaccharide
miRNA: MicroRNA
NF-κB: Nuclear factor kappa B
RT-PCR: Real-time polymerase chain reaction
VEGF: Vascular endothelial growth factor

## References

[1] Bone RC, Balk RA, Cerra FB, Dellinger RP, Fein AM, Knaus WA (1992). Definitions for sepsis and organ failure and guidelines for the use of innovative therapies in sepsis. The ACCP/SCCM Consensus Conference Committee. American College of Chest Physicians/Society of Critical Care Medicine. Chest.

[2] Angus DC, van der Poll T (2013). Severe sepsis and septic shock. N Engl J Med..

[3] Aird WC (2003). The role of the endothelium in severe sepsis and multiple organ dysfunction syndrome. Blood..

[4] Faure E, Thomas L, Xu H, Medvedev A, Equils O, Arditi M (2001). Bacterial lipopolysaccharide and IFN-gamma induce Toll-like receptor 2 and Toll-like receptor 4 expression in human endothelial cells: role of NF-kappa B activation. J Immunol..

[5] Henneke P, Golenbock DT (2002). Innate immune recognition of lipopolysaccharide by endothelial cells. Crit Care Med.

[6] Zhang FX, Kirschning CJ, Mancinelli R, Xu XP, Jin Y, Faure E (1999). Bacterial lipopolysaccharide activates nuclear factor-kappaB through interleukin-1 signaling mediators in cultured human dermal endothelial cells and mononuclear phagocytes. J Biol Chem..

[7] Lee RC, Feinbaum RL, Ambros V (1993). The C. elegans heterochronic gene lin-4 encodes small RNAs with antisense complementarity to lin-14. Cell.

[8] Lee RC, Ambros V (2001). An extensive class of small RNAs in Caenorhabditis elegans. Science..

[9] Lagos-Quintana M, Rauhut R, Lendeckel W, Tuschl T (2001). Identification of novel genes coding for small expressed RNAs. Science..

[10] Valadi H, Ekstrom K, Bossios A, Sjostrand M, Lee JJ, Lotvall JO (2007). Exosome-mediated transfer of mRNAs and microRNAs is a novel mechanism of genetic exchange between cells. Nat Cell Biol..

[11] Zernecke A, Bidzhekov K, Noels H, Shagdarsuren E, Gan L, Denecke B (2009). Delivery of microRNA-126 by apoptotic bodies induces CXCL12-dependent vascular protection. Sci Signal.

[12] Hergenreider E, Heydt S, Treguer K, Boettger T, Horrevoets AJ, Zeiher AM (2012). Atheroprotective communication between endothelial cells and smooth muscle cells through miRNAs. Nat Cell Biol..

[13] Mittelbrunn M, Gutierrez-Vazquez C, Villarroya-Beltri C, Gonzalez S, Sanchez-Cabo F, Gonzalez MA (2011). Unidirectional transfer of microRNA-loaded exosomes from T cells to antigen-presenting cells. Nat Commun..

[14] Fan H, Goodwin AJ, Chang E, Zingarelli B, Borg K, Guan S (2014). Endothelial progenitor cells and a stromal cell-derived factor-1alpha analogue synergistically improve survival in sepsis. Am J Respir Crit Care Med..

[15] Fish JE, Santoro MM, Morton SU, Yu S, Yeh RF, Wythe JD (2008). miR-126 regulates angiogenic signaling and vascular integrity. Dev Cell.

[16] Wang S, Aurora AB, Johnson BA, Qi X, McAnally J, Hill JA (2008). The endothelial-specific microRNA miR-126 governs vascular integrity and angiogenesis. Dev Cell..

[17] Harris TA, Yamakuchi M, Ferlito M, Mendell JT, Lowenstein CJ (2008). MicroRNA-126 regulates endothelial expression of vascular cell adhesion molecule 1. Proc Natl Acad Sci U S A..

[18] Wang X, Wang X, Liu X, Wang X, Xu J, Hou S (2015). miR-15a/16 are upreuglated in the serum of neonatal sepsis patients and inhibit the LPS-induced inflammatory pathway. Int J Clin Exp Med.

[19] Wang H, Zhang P, Chen W, Feng D, Jia Y, Xie LX (2012). Evidence for serum miR-15a and miR-16 levels as biomarkers that distinguish sepsis from systemic inflammatory response syndrome in human subjects. Clin Chem Lab Med..

[20] Acosta-Herrera M, Lorenzo-Diaz F, Pino-Yanes M, Corrales A, Valladares F, Klassert TE (2015). Lung transcriptomics during protective ventilatory support in sepsis-induced acute lung injury. PLoS One..

[21] Wang Z, Ruan Z, Mao Y, Dong W, Zhang Y, Yin N (2014). miR-27a is up regulated and promotes inflammatory response in sepsis. Cell Immunol.

[22] Roderburg C, Luedde M, Vargas Cardenas D, Vucur M, Scholten D, Frey N (2013). Circulating microRNA-150 serum levels predict survival in patients with critical illness and sepsis. PLoS One..

[23] Ma Y, Vilanova D, Atalar K, Delfour O, Edgeworth J, Ostermann M (2013). Genome-wide sequencing of cellular microRNAs identifies a combinatorial expression signature diagnostic of sepsis. PLoS One..

[24] Wang JF, Yu ML, Yu G, Bian JJ, Deng XM, Wan XJ (2010). Serum miR-146a and miR-223 as potential new biomarkers for sepsis. Biochem Biophys Res Commun..

[25] Wang HJ, Zhang PJ, Chen WJ, Feng D, Jia YH, Xie LX (2012). Four serum microRNAs identified as diagnostic biomarkers of sepsis. J Trauma Acute Care Surg..

[26] Sun X, Icli B, Wara AK, Belkin N, He S, Kobzik L (2012). MicroRNA-181b regulates NF-kappaB-mediated vascular inflammation. J Clin Invest..

[27] Tili E, Michaille JJ, Cimino A, Costinean S, Dumitru CD, Adair B (2007). Modulation of miR-155 and miR-125b levels following lipopolysaccharide/TNF-alpha stimulation and their possible roles in regulating the response to endotoxin shock. J Immunol..

[28] Taganov KD, Boldin MP, Chang KJ, Baltimore D (2006). NF-kappaB-dependent induction of microRNA miR-146, an inhibitor targeted to signaling proteins of innate immune responses. Proc Natl Acad Sci U S A..

[29] Song L, Lin C, Gong H, Wang C, Liu L, Wu J (2013). miR-486 sustains NF-kappaB activity by disrupting multiple NF-kappaB-negative feedback loops. Cell Res.

[30] McClure C, Brudecki L, Ferguson DA, Yao ZQ, Moorman JP, McCall CE (2014). MicroRNA 21 (miR-21) and miR-181b couple with NFI-A to generate myeloid-derived suppressor cells and promote immunosuppression in late sepsis. Infect Immun..

[31] Barnett RE, Conklin DJ, Ryan L, Keskey RC, Ramjee V, Sepulveda EA (2015). Anti-inflammatory effects of miR-21 in the macrophage response to peritonitis. J Leukoc Biol..

[32] Force ADT, Ranieri VM, Rubenfeld GD, Thompson BT, Ferguson ND, Caldwell E (2012). Acute respiratory distress syndrome: the Berlin Definition. JAMA..

[33] Medina RJ, O'Neill CL, O'Doherty TM, Wilson SEJ, Stitt AW (2012). Endothelial Progenitors as Tools to Study Vascular Disease. Stem Cells Int..

[34] TargetScanHuman: Prediction of microRNA targets. Available from: http://www.targetscan.org/. Accessed 16 July 2015

[35] microRNA.org - Targets and Expression. Available from: http://www.microrna.org/microrna/home.do. Accessed 16 July 2015.

[36] Miller TE, Ghoshal K, Ramaswamy B, Roy S, Datta J, Shapiro CL (2008). MicroRNA-221/222 confers tamoxifen resistance in breast cancer by targeting p27Kip1. J Biol Chem..

[37] Takagi T, Naito Y, Mizushima K, Hirata I, Yagi N, Tomatsuri N (2010). Increased expression of microRNA in the inflamed colonic mucosa of patients with active ulcerative colitis. J Gastroenterol Hepatol..

[38] Thery C, Ostrowski M, Segura E (2009). Membrane vesicles as conveyors of immune responses. Nat Rev Immunol..

[39] Arroyo JD, Chevillet JR, Kroh EM, Ruf IK, Pritchard CC, Gibson DF (2011). Argonaute2 complexes carry a population of circulating microRNAs independent of vesicles in human plasma. Proc Natl Acad Sci U S A..

[40] Vickers KC, Palmisano BT, Shoucri BM, Shamburek RD, Remaley AT (2011). MicroRNAs are transported in plasma and delivered to recipient cells by high-density lipoproteins. Nat Cell Biol..

[41] Gavard J, Gutkind JS (2006). VEGF controls endothelial-cell permeability by promoting the beta-arrestin-dependent endocytosis of VE-cadherin. Nat Cell Biol..

[42] van der Flier M, van Leeuwen HJ, van Kessel KP, Kimpen JL, Hoepelman AI, Geelen SP (2005). Plasma vascular endothelial growth factor in severe sepsis. Shock..

[43] Pickkers P, Sprong T, Eijk L, Hoeven H, Smits P, Deuren M (2005). Vascular endothelial growth factor is increased during the first 48 hours of human septic shock and correlates with vascular permeability. Shock..

[44] Gao L, Grant A, Halder I, Brower R, Sevransky J, Maloney JP (2006). Novel polymorphisms in the myosin light chain kinase gene confer risk for acute lung injury. Am J Respir Cell Mol Biol..

[45] Ackermann EJ, Taylor JK, Narayana R, Bennett CF (1999). The role of antiapoptotic Bcl-2 family members in endothelial apoptosis elucidated with antisense oligonucleotides. J Biol Chem..

[46] Potente M, Dimmeler S (2008). Emerging roles of SIRT1 in vascular endothelial homeostasis. Cell Cycle..

[47] Pan Y, Liang H, Liu H, Li D, Chen X, Li L (2014). Platelet-secreted microRNA-223 promotes endothelial cell apoptosis induced by advanced glycation end products via targeting the insulin-like growth factor 1 receptor. J Immunol..

[48] Pritchard CC, Kroh E, Wood B, Arroyo JD, Dougherty KJ, Miyaji MM (2012). Blood cell origin of circulating microRNAs: a cautionary note for cancer biomarker studies. Cancer Prev Res (Phila).

[49] Guduric-Fuchs J, O'Connor A, Camp B, O'Neill CL, Medina RJ, Simpson DA (2012). Selective extracellular vesicle-mediated export of an overlapping set of microRNAs from multiple cell types. BMC Genomics..

[50] Pigati L, Yaddanapudi SC, Iyengar R, Kim DJ, Hearn SA, Danforth D (2010). Selective release of microRNA species from normal and malignant mammary epithelial cells. PLoS One..

[51] Yin KJ, Olsen K, Hamblin M, Zhang J, Schwendeman SP, Chen YE (2012). Vascular endothelial cell-specific microRNA-15a inhibits angiogenesis in hindlimb ischemia. J Biol Chem..

[52] Goretti E, Rolland-Turner M, Leonard F, Zhang L, Wagner DR, Devaux Y (2013). MicroRNA-16 affects key functions of human endothelial progenitor cells. J Leukoc Biol..

[53] Ito T, Yagi S, Yamakuchi M (2010). MicroRNA-34a regulation of endothelial senescence. Biochem Biophys Res Commun..

